# Recent advances in understanding of attention deficit hyperactivity disorder (ADHD): how genetics are shaping our conceptualization of this disorder

**DOI:** 10.12688/f1000research.18959.2

**Published:** 2020-02-12

**Authors:** Tetyana Zayats, Benjamin M Neale

**Affiliations:** 1 Analytic and Translational Genetics Unit, Massachusetts General Hospital and Harvard Medical School, Boston, MA, 02114, USA; 2 Stanley Center for Psychiatric Research, Broad Institute of MIT and Harvard, Cambridge, MA, 02142, USA

**Keywords:** Attention deficit hyperactivity disorder, ADHD, genetic predisposition, Genome-wide association studies, Pharmacogenetics, genetics

## Abstract

Attention deficit hyperactivity disorder (ADHD) is a clinically defined disorder, and inattention and hyperactivity/impulsivity are its main symptom domains. The presentation, lifelong continuation and treatment response of ADHD symptoms, however, is highly heterogeneous. To better define, diagnose, treat and prevent ADHD, it is essential that we understand the biological processes underlying all of these elements. In this review, given the high heritability of ADHD, we discuss how and why genetics can foster such progress. We examine what genetics have taught us so far with regard to ADHD definition, classification, clinical presentation, diagnosis and treatment. Finally, we offer a prospect of what genetic studies on ADHD may bring in the future.

## Introduction

Attention deficit hyperactivity disorder (ADHD) is a clinically defined disorder, and inattention and hyperactivity/impulsivity are its main symptom domains ^1^. The presentation, life-long continuation and treatment response of ADHD symptoms, however, is highly heterogeneous, underscored by the wide array of psychiatric and somatic comorbidities.

To better define, diagnose, treat and prevent ADHD, it is essential that we understand the biological processes underlying all of these elements. As family and twin studies revealed that genetics contribute to the etiology of ADHD (heritability estimates range from 60 to 90% ^1, 2^), the conceptualization of this disorder moved away from being a consequence of early brain damage to being a multifactorial phenotype, with both genetics and environment affecting its development, trajectory and outcome. Here, we discuss why and how the recent genetic findings on ADHD may shape our understanding of its definition, diagnosis, treatment and prevention.

## Why study genetics in attention deficit hyperactivity disorder?

The view of ADHD as a multifactorial disorder with a genetic component comes from the clinical complexity observed in ADHD’s symptomatology. ADHD runs in families and co-occurs in identical twins at a much higher rate than in fraternal twins ^3, 4^. This familial aggregation suggests that genetics can serve as a tool to identify the main biological drivers behind ADHD development as well as its lifetime trajectory. Furthermore, genetics can be used to probe the genetic overlap between ADHD and various psychiatric and somatic disorders and traits. These kinds of analyses can aid the definition and classification of this disorder and lead to a better understanding of its comorbidity. Evaluation of the degree of genetic susceptibility to ADHD phenotypes can help the establishment of genetic counselling today and, in the future, lead to improved evaluation of prognosis and provision of effective treatment options that act at the etiological level of ADHD. This notion was recently affirmed by a genome-wide association (GWA) study that revealed the first genome-wide significant loci associated with ADHD ^5^, offering possibilities to further our understanding of this disorder. Perhaps the most important one is that ADHD appears to be a disorder of central nervous system–specific regulatory elements.

## What have we learned from genetics so far?

### Definition and classification

Traditionally, ADHD has been classified as an externalizing behavioral disorder. However, as genetic epidemiological studies have shown high familial overlap between ADHD and autism spectrum disorder (ASD) and between ADHD and intellectual disability (ID), the classification shifted toward neurodevelopmental disorders ^2^. This notion has recently been further affirmed by observations of ADHD displaying genetic correlation and overlap with ASD at the levels of both common and rare genetic variation ^6, 7^. In addition, common genetic factors have been shown to contribute to the overall correlation between ADHD and ID (except for profound ID) ^8^. Another observation in favor of ADHD being a neurodevelopmental disorder is the higher prevalence of ADHD among boys compared to girls ^9^. Nonetheless, ADHD also shows genetic overlap with behavioral problems ^5, 10, 11^, and recent genetic study notes that common genetic variation may not explain the sex differences in its diagnosis ^12^, suggesting that the clear-cut classification of ADHD is still an open question.

Also, traditionally, ADHD has been defined as a unitary disorder with a number of subtypes (that is, inattentive, hyperactive and combined subtypes). One way to evaluate such a definition is to explore the notion that ADHD cases may be defined as extremes of the distribution of ADHD symptoms (both inattention and hyperactivity/impulsivity) ^2, 5, 11^, much in the way that hypertension is defined to be the extreme end of blood pressure distribution in a population. To define ADHD in that fashion, we must consider whether ADHD symptoms (that is, inattention and hyperactivity/impulsivity) display consistent co-occurrence with sufficient degree of intensity and duration to form a biologically and clinically meaningful entity of ADHD. The early (and under-powered) GWA studies of these traits revealed both unique and shared genetic influences on these dimensions of ADHD ^13, 14^. A more recent GWA study of these traits, in more than 37,500 children, showed high genetic correlation between continuous measures of inattention and hyperactivity in children (rg = 73%) and between those two traits and ADHD diagnosis in both children and adults (rg _inattention+ADHD_ = 93%, rg _hyperactivity/impulsivity+ADHD_ = 91%). The exploration of genetic correlations of these dimensions with common psychiatric disorders and traits revealed two distinct patterns of correlations—inattention correlated more with neurodevelopmental phenotypes and hyperactivity/impulsivity correlated more with behavioral problems ^15^—highlighting the dual nature of ADHD as it is defined today. Thus, on the basis of the recent genetic studies, ADHD may be defined not as a unitary disorder with several subtypes but rather as a spectrum disorder whose core symptoms (inattention or hyperactivity/impulsivity or both) interfere with an individual’s functioning in important life aspects. In fact, such change in conceptualization of ADHD may already be seen in the latest version of the *Diagnostic and Statistical Manual of Mental Disorders* (DSM), where the three ADHD “subtypes” have been substituted by three ADHD “representations” (potentially also reflecting the fluidity of the ADHD symptomatology over a life span) ^16^.

### Diagnosis and clinical representation

The diagnosis of ADHD relies heavily on how we define it. The two diagnostic systems of contemporary psychiatry—the International Statistical Classification of Diseases and Related Health Problems (currently, ICD-10 ^17^) and the DSM (currently, DSM-5 ^16^)—base a clinical diagnosis of ADHD (or hyperkinetic disorder (HKD) in ICD-10) on the two sets of symptom domains: inattention and hyperactivity/impulsivity. Although ICD-10 and DSM-5 operate with the same two symptom domains to define ADHD/HKD, the diagnosis of ASD or bipolar disorder precludes the diagnosis of HKD in ICD-10, whereas DSM-5 does allow the presence of diagnoses of both ADHD and ASD. The diagnostic criterion of ICD-10 is in direct conflict with recent findings that ADHD and ASD do have a common genetic (and possibly etiological) component ^6, 7^. This highlights the recent perception that the current diagnostic scheme for ADHD (and many other major psychiatric disorders) is not reflective of its underlying biological foundation and that the eventual goal is to move away from clinically defined diagnoses to molecularly defined ones ^18, 19^.

Reflecting the view of ADHD as an extreme on the continuum of its two main domains (inattention and hyperactivity/impulsivity), the diagnosis of ADHD faces the questions of which symptoms to consider and to what extent. In ICD-10, for example, the HKD is a unity of symptoms (all three sets of symptoms must be present to diagnose ADHD), all symptoms must be exhibited in more than one setting (for example, home and school) and the presence of comorbidities is practically not allowed. In contrast, the DSM-5 distinguishes three different diagnostic ADHD presentations (not all three sets of symptoms must be present in order to diagnose ADHD), the symptoms need to be present in only some settings and the presence of comorbidities is freely allowed (as exemplified by ASD above). Given this discordant view of ADHD diagnosis between the two major diagnostic systems and given that recent genetic studies on ADHD revealed that it exhibits an extensive genetic overlap with a wide range of psychiatric disorders ^5, 11^, the two main symptom domains of ADHD may be a non-specific component in a variety of conditions and the diagnosis of ADHD may be a quantitative rather than a qualitative entity.

### Treatment

It has been reported that the current pharmacological ADHD treatment is effective in about 70% of cases ^20^. The major obstacle to developing a more effective treatment for ADHD is our limited understanding of what causes the disorder and the mechanism (or mechanisms) through which the current pharmaceuticals are acting on ADHD. The barriers to progress are many and varied, but the inaccessibility of live human brain tissues makes progress in the neurobiological basis of ADHD particularly challenging. One option to circumvent this challenge is to use induced pluripotent stem cells that could provide a promising avenue for downstream molecular interrogation of genome-wide significant loci ^21^. Although arguably a clinician could treat a disorder without understanding it, we must make a distinction between symptom alleviation and a cure. Currently, all of the existing treatment options for ADHD (both pharmacological and behavioral) offer symptomatic relief only ^22^.

With the recent technological advances and large collaborative efforts, more and more large-scale GWA studies are becoming available on a variety of somatic and psychiatric phenotypes, including ADHD. These studies are an important source of information for the rapidly evolving field of ADHD pharmacogenetics ^23, 24^ that may help to circumvent the current limitations of drug development and re-purposing. Using data from the first well-powered GWA study on ADHD ^5^, the examination of the association between ADHD and the genes encoding the targets of the first-line US Food and Drug Administration (FDA)-approved pharmacological agents for ADHD treatment revealed no significant findings ^22^, suggesting that those pharmaceuticals may act through mechanisms other than the ones underlying ADHD (although currently the largest ADHD GWA study still does not capture the biology of ADHD in its entirety).

The current FDA-approved treatments for ADHD are primarily thought to enhance catecholamine signaling. However, such a narrow pharmacological target stands in contrast to the complexity of emerging genetic findings, which suggest that other avenues of therapeutic intervention may be possible. As we learn more about the biological basis of ADHD, these findings could enable the development of new drugs through different mechanisms of actions. Furthermore, drug re-purposing of already-approved compounds and treatments may be a faster path to improving the quality of care for patients. One way to nominate such potential treatments might be to evaluate treatment options for traits with high genetic correlation to ADHD, motivating the systematic evaluation of genetic overlap between ADHD and other phenotypes ^25^.

A potentially successful example of drug re-purposing guided by genetic studies of ADHD is the trial use of fasoracetam as a treatment for this disorder. Originally developed as pharmacotherapy for vascular dementia, fasoracetam has been successfully used in a clinical trial to treat ADHD in adolescents with disrupted glutamatergic signaling that has been shown to be associated with ADHD ^26^.

Although the re-purposing and development of new pharmacotherapeutics for ADHD takes time, it is important to note that the mere shift in understanding of ADHD as a multifactorial disorder with a genetic component may help patients in their management of the disorder ^27^.

### Diagnostic screening and prevention

To reliably screen individuals for ADHD on the basis of common genetic variants, we first need to establish the true effect sizes of the variants associated with the disorder. So far, only one relatively well-powered GWA study on ADHD has provided estimates of these effects ^5^, but those estimates are not accurate enough for diagnostic purposes in clinical settings. As the power of genetic studies improves, the assessment of the number and the effect sizes of genetic variants robustly associated with ADHD will also improve, increasing the potential of common variants to become a helpful tool in a clinical setting, much in the way that polygenic risk score (PRS) is used in coronary heart disease ^28– 30^. In the meantime, although the diagnostic usefulness of common genetic variants is still far from reality for ADHD, the genetic profile of a cumulative number of ADHD risk alleles (PRS) can be of benefit for patients whose ADHD has already been diagnosed as, in the near future, PRS is more likely to aid the prognosis of ADHD, especially in combination with additional non-genetic information (for example, family history).

The clinical utility of rare genetic variants, in contrast to that of common ones, tends to be stronger as their penetrance (that is, the chance of developing the disorder) tends to be much higher. However, despite recent studies showing genetic overlap between ADHD and neurodevelopmental disorders ^7, 8, 31^, there is little evidence to support the need for genetic testing based on rare variants, especially as none of those variants is ADHD-specific.

## What can genetics of attention deficit hyperactivity disorder tell us in the future?

Following in footsteps of the first genome-wide significant ADHD loci discovery, we must next replicate and understand these findings. GWA studies in independent large(r) samples are expected to shed light on the validity of these loci and examination of their functionality will aid our understanding of biological processes underlying ADHD. Thus, further work at the molecular level of neural cells, systems and circuits can be anticipated from both bioinformatics and experimental systems biology.

There is a growing interest in investigation of ADHD across the life span as it has been noted that persistence of ADHD symptoms is associated with a high number of genetic ADHD risk variants that an individual may possess ^32^. As large phenotypically informative and genotyped cohorts become more available, it will be possible to address questions of biological background of ADHD continuation throughout life (for example, longitudinal studies) and determine periods critical for the development, lifelong trajectory and treatment of this disorder.

In addition, such cohorts will allow the examination of the causal impact of loci associated with ADHD that may help elucidate the reasons behind high correlations between ADHD and a wide range of psychiatric and somatic disorders and traits.

One branch of genetics that has received little attention in ADHD so far is the examination of direct and indirect (environmental) genetic effects influencing ADHD. To date, all genome-wide genetic studies on ADHD, except for one carried out by Wang and colleagues ^33^, assumed that this disorder can be influenced only by the genetics of an individual with ADHD (direct genetic effects). However, the expression of a phenotype in an individual is influenced not only by their own genotype (direct genetic effect) but also by the genotype of people in their environment, such as their mother, father, or siblings (indirect genetic effects) ^34^. The evaluation of environment’s role in the development of ADHD could also benefit from gene–environment interaction studies. However, probing the environmental effects in ADHD is often limited by gene–environment correlation where the association between ADHD and an environmental factor can be the result of inherited confounds ^35^. The disentangling of these direct and indirect (environmental) effects has the potential to advance our understanding of such long-standing observations as missing heritability (the difference in heritability estimates between genetic and epidemiological studies), sex differences in ADHD prevalence, variability in persistence of ADHD symptoms across a life span and non-Mendelian forms of ADHD inheritance and aid in ADHD prevention and treatment.

Finally, the recently evolving branches of genetics can also elucidate the pharmacology of ADHD (pharmacogenetics) and environmental effects critical for clinical aspects of ADHD (geno-economics, geno-epidemiology, epigenetics, and parent-of-origin effects).
